# Characterization of *In Vitro* Engineered Human Adipose Tissues: Relevant Adipokine Secretion and Impact of TNF-α

**DOI:** 10.1371/journal.pone.0137612

**Published:** 2015-09-14

**Authors:** Kim Aubin, Meryem Safoine, Maryse Proulx, Marie-Alice Audet-Casgrain, Jean-François Côté, Félix-André Têtu, Alphonse Roy, Julie Fradette

**Affiliations:** 1 Centre de recherche en organogénèse expérimentale de l’Université Laval / LOEX, Québec, Canada; 2 Division of Regenerative Medicine, CHU de Québec Research Centre, Québec, Canada; 3 Clinique de chirurgie esthétique Félix-André Têtu and CHU de Québec, Québec, Canada; 4 Clinique de chirurgie plastique Alphonse Roy and CHU de Québec, Québec, Canada; 5 Department of Surgery, Faculty of Medicine, Université Laval, Québec, Canada; University of Western Ontario, CANADA

## Abstract

Representative modelling of human adipose tissue functions is central to metabolic research. Tridimensional models able to recreate human adipogenesis in a physiological tissue-like context *in vitro* are still scarce. We describe the engineering of white adipose tissues reconstructed from their cultured adipose-derived stromal precursor cells. We hypothesize that these reconstructed tissues can recapitulate key functions of AT under basal and pro-inflammatory conditions. These tissues, featuring human adipocytes surrounded by stroma, were stable and metabolically active in long-term cultures (at least 11 weeks). Secretion of major adipokines and growth factors by the reconstructed tissues was determined and compared to media conditioned by human native fat explants. Interestingly, the secretory profiles of the reconstructed adipose tissues indicated an abundant production of leptin, PAI-1 and angiopoietin-1 proteins, while higher HGF levels were detected for the human fat explants. We next demonstrated the responsiveness of the tissues to the pro-inflammatory stimulus TNF-α, as reflected by modulation of MCP-1, NGF and HGF secretion, while VEGF and leptin protein expression did not vary. TNF-α exposure induced changes in gene expression for adipocyte metabolism-associated mRNAs such as *SLC2A4*, *FASN* and *LIPE*, as well as for genes implicated in NF-κB activation. Finally, this model was customized to feature adipocytes representative of progressive stages of differentiation, thereby allowing investigations using newly differentiated or more mature adipocytes. In conclusion, we produced tridimensional tissues engineered *in vitro* that are able to recapitulate key characteristics of subcutaneous white adipose tissue. These tissues are produced from human cells and their neo-synthesized matrix elements without exogenous or synthetic biomaterials. Therefore, they represent unique tools to investigate the effects of pharmacologically active products on human stromal cells, extracellular matrix and differentiated adipocytes, in addition to compounds modulating adipogenesis from precursor cells.

## Introduction

Adipose tissue (AT) is a highly active organ that regained particular attention considering its contributions to obesity-related dysfunctions such as insulin resistance and cardiovascular diseases [1–3]. In fact, white AT (WAT) predominates in humans, with distinct metabolic contributions of the visceral depots compared to the subcutaneous ones, especially under conditions of weight gain and obesity [4, 5]. In addition, recent descriptions of brown as well as beige/brite adipocytes in adults spurred a strong interest for these highly thermogenic cells of distinct developmental origin [6, 7]. WAT depots produce a great variety of active mediators that are secreted into the circulation, therefore impacting on many cell types and tissues [8]. Adipocytes, stromal cells and other resident cells (endothelial, immune cells, etc.) all contribute to AT secretome and functions, which are not restricted to fatty acid metabolism but also influence processes such as inflammation, immune modulation and angiogenesis [9, 10]. In particular, AT secreted factors such as leptin, plasminogen activator inhibitor-1 (PAI-1), angiopoietin-1 (Ang-1), vascular endothelial growth factor (VEGF) and hepatocyte growth factor (HGF) can impact vascular networks by acting on endothelial cell proliferation, migration and permeability. Ang-1 and PAI-1 are also known for their ability to influence capillary stability and the coordination between the adipogenic and angiogenic processes occurring during AT expansion [11–16].

Low-grade chronic inflammation characterizes the obese state and therefore, adipocytes are in contact with potent inflammatory mediators such as TNF-α (tumor necrosis factor α) and IL-1β (interleukin-1β) [17–19]. TNF-α affects many biological processes including differentiation, apoptosis and energy metabolism, in addition to modulating inflammation [3, 20]. Adipocytes are also producers of various interleukins such as IL-6, IL-8, IL-10 and IL-1β, therefore contributing to the balance between pro- and anti-inflammatory networks [21, 22]. In fact, a novel research area termed immunometabolism has emerged from the study of AT’s roles in metabolism and immunity, which contribute to the pathogenesis of obesity-associated dysfunctions [23, 24]. It is of high importance to dissect adipose tissue responses during inflammation and pathological situations. For such studies, the use of relevant human adipose tissue models engineered *in vitro* could greatly contribute to the field.

Adipocytes develop from mesenchymal precursors through a coordinated adipogenic program that was initially discovered using the immortalized rodent cultures 3T3-L1 and 3T3-F442A [25, 26]. This pioneering work established the molecular basis of adipogenesis, and differentiated 3T3-L1 and 3T3-F442A adipocytes are still widely used to study adipocyte metabolism considering that they are easily cultured compared to the rather short-lived isolated primary adipocytes or organotypic cultures [27, 28]. Of course, the human adipogenic program can also be recapitulated using human mesenchymal precursors cells. In particular, the heterogeneous stromal-vascular (SVF) fraction resulting from the enzymatic dissociation of subcutaneous AT has been much studied for its therapeutic potential in recent years [29, 30]. This is due to the fact that once plated and amplified in culture, adherent SVF cells are then called adipose-derived stem/stromal cells *(*ASCs), which have been shown to contain not only preadipocytes but also a subpopulation of stromal cells endowed with multilineage differentiation capacity including neuronal cells, chondrocytes and osteoblasts [31, 32]. Moreover, an important part of the therapeutic effects mediated by mesenchymal stem cells such as ASCs are associated with their secretion of cytokines and growth factors promoting regenerative processes through cell recruitment and proliferation while limiting apoptosis [33, 34].

Recent developments in the field of tissue engineering expanded the range of tridimensional (3D) models available as research tools. Engineering of adipose tissue is mainly driven by the need to develop human substitutes intended to restore soft tissue defects during reconstructive or cosmetic procedures, given the variable long-term success rates of the current autologous fat grafting procedures [35]. Due to their 3D structure, engineered adipose tissues also represent excellent *in vitro* models for studying adipocytes under physiological or pathological conditions since they usually feature a matrix-rich tissue-like context recapitulating the niche. Extracellular matrix (ECM) components have proven to be essential for proper cell proliferation, differentiation and signaling [36, 37]. Among models that are being developed for AT engineering, the use of synthetic, biomimetic and natural matrices such as decellularized tissues has been tested in conjunction with cells of various origin and species [38]. Our team has previously shown that ASCs extracted from lipoaspirated fat can be expanded in culture and used as building blocks for tissue engineering applications using the self-assembly approach. This method consists of stimulating ECM production/deposition by the cells themselves with ascorbic acid. By concomitantly inducing adipogenic differentiation *in vitro*, the resulting adipose cell sheets feature numerous adipocytes as well as natural ECM components [39]. The production of thicker tissues can be customized through the superposition of many cell sheets. In this study, our goal was to determine if these *in vitro* human reconstructed adipose tissues (hrAT) could recapitulate specific AT functions under basal conditions and in response to a pro-inflammatory stimulus (TNF-α). To do so, we performed a detailed characterization of these hrAT samples. The long-term maintenance and viability in culture of these adipose substitutes was assessed by following adipocyte development and secreted factors production. Comparative data for leptin, Ang-1, VEGF, PAI-1 and HGF release in conditioned media from hrAT as well as native human AT explants provided information on the shared contribution of adipocytes and stromal cells to the secretome. Finally, the responsiveness of the engineered tissues to the classic pro-inflammatory cytokine TNF-α, both at the gene and protein expression levels, confirmed the relevance of this 3D model as a novel tool to investigate biological responses of human WAT.

## Materials and Methods

### Ethics Statement

All studies involving human tissues and cells in culture were specifically approved by the Ethics Committee of the CHU de Québec Research Centre (# DR-002-1117). Human subcutaneous AT was obtained from non-obese men and women undergoing lipoaspiration or lipectomy procedures, following their written informed consent for the use of these samples in research.

### Tissues and culture systems

The characteristics of donors and AT samples used for organotypic cultures as well as for tissue reconstruction are described in Table 1. Freshly harvested ATs were rinsed with phosphate-buffered saline (PBS) solution containing antibiotics [100 U/ml penicillin (Sigma-Aldrich, Oakville, ON, Canada) and 25 μg/ml gentamicin (Schering-Plough Canada Inc./Merck, Scarborough, ON, Canada)]. Organotypic cultures were established by placing AT fragments (average explants weight 851.6 mg, Table 1) in 24-well plates (VWR, Mississauga, ON, Canada) containing 1.5 ml of complete adipocyte maintenance medium consisting of basal DMEM: F-12 (1:1) (DH) supplemented with 10% fetal calf serum (FCS, HyClone, Logan, UT, USA), 100 nM insulin (Sigma), 0.2 nM T3 (Sigma), 1 μM dexamethasone (Sigma) and antibiotics. Explants were incubated for 48 h at 37°C in a humidified atmosphere containing 8% CO_2_. Controls for protein quantification consisted of media alone incubated under the same conditions. Conditioned media were harvested and frozen at -80°C until analysis. Explants were quick-frozen in liquid nitrogen for DNA content determination. ASC cultures were established from lipoaspirates and frozen at passage 0 according to a previously described methodology [39]. Cells were thawed and expanded in DH medium supplemented with 10% FCS and antibiotics.

**Table 1:** Description of AT used for organotypic cultures and/or tissue reconstruction.

**Population ID number**	**Age of donor**	**BMI of donor**	Mean weight (mg)*	**Number of samples (n)**	**Analyses performed on samples**
**AT explants**					
1	32	23.3	610.0 (76.7)	6	Basal secretion
2	35	21.0	1807.7 (380.5)	3	Basal secretion
3 ^#^	36	25.0	870.2 (136.2)	3	Basal secretion
4	38	20.1	467.4 (120.8)	6	Basal secretion
5 ^&^	41	28.8	867.2 (70.1)	4	Basal secretion
6 ^#^ ^,^ ^&^	55	25.3	486.9 (100.3)	6	Basal secretion
**Mean (SD)**	**39.5 (8.2)**	**23.9 (3.2)**	**851.6 (500.7)**	**-**	**-**
	51	22.0	806.2 (123.8)	3	10 ng/ml TNF-α 24 h- treatment
7	51	22.0	791.7 (52.0)	3	Control, non-treated samples
	58	24.6	233.6 (35.0)	3	10 ng/ml TNF-α 24 h- treatment
8	58	24.6	232.1 (11.1)	3	Control, non-treated samples
**Mean (SD)**	**54.5 (4.9)**	**23.3 (1.8)**	**515.9 (326.9)**	**-**	**-**
**Population ID number**	**Age of donor**	**BMI of donor**	Mean weight (mg)* hrAT / hrCT	**Number of samples (n) hrAT / hrCT**	**Analyses performed on samples**
**Reconstructed tissues (3.5 cm** ^**2**^ **)**					
3 ^#^	36	25.0	30.1 (1.8) / 31.8 (1.3)	2 / 3	Basal secretion
6 ^#^ ^,^ ^&^	55	25.3	29.6 (2.2) / 26.1 (0.5)	3 / 3	Basal secretion
9	38	29.5	28.1 (0.7) / 39.1 (0.9)	2 / 3	Basal secretion
10	54	24.9	26.3 (1.9) / 18.5 (1.7)	3 / 3	Basal secretion
**Mean (SD)**	**45.8 (10.1)**	**26.2 (2.2)**	**28.5 (1.7) / 28.9 (8.7)**	**-**	**-**

*: Values are indicated as mean (SD) when applicable.

&: indicates tissues from male donors.

#: indicates data available for both AT explants and tissues reconstructed using the cells extracted from adipose tissue from the same donor.

### Production of reconstructed sheets and tissues

The self-assembly approach of tissue engineering was used to produce ASC-based connective or adipose cell sheets [39]. After expansion, ASCs were seeded at passage 3 in DH medium supplemented with 10% FCS and antibiotics at a density of 1.56 x 10^4^ cell/cm^2^ in Nunc 6-well plates (Thermo Fisher Scientific, Waltham, MA, USA) containing paper anchorage devices (Whatman, Fisher Scientific, Ottawa, ON, Canada) to produce cell sheets of 3.5 cm^2^ surface area. Cultures were supplemented with ascorbic acid (AsA) (Sigma-Aldrich, Oakville, ON, Canada) freshly prepared at each media change (every 2–3 days) and used at a concentration of 50 μg/ml (250 μM) throughout the culture period. If not specified otherwise, adipogenic induction was performed after 7 days of culture by using a defined cocktail containing 100 nM insulin (Sigma), 0.2 nM T3 (Sigma), 1 μM dexamethasone (Sigma), 0.25 mM 3-isobutyl-1-methylxanthine (IBMX, Sigma) and 1 μM rosiglitazone (Cayman Chemical/Cedarlane, Burlington, ON, Canada) in 3% FCS-containing medium supplemented with AsA. After 3 days of induction, culture was continued using complete adipocyte maintenance medium supplemented with AsA for the rest of the culture period [39, 40]. In parallel, reconstructed connective sheets were produced from the same ASC populations, by omitting the induction step (mock control media containing 3% FCS and 0.038% dimethyl sulfoxide) and further culturing in 10% FCS DH medium (with AsA and antibiotics). After 28 days of culture, thicker human reconstructed connective tissues (hrCT) and hrAT were produced by the superposition of three individual cell sheets, and further cultured for at least 7 days before being harvested for analyses. Finally, an additional protocol for the engineering of hrAT was evaluated in order to obtain adipocytes at specific stages of differentiation. Static cultures were compared to dynamic culture conditions created by a 3D shaker platform gyrating at 35 rpm (GyroTwister™ and Ocelot Rotator, Fisher Scientific) inducing a wave-like motion of the medium throughout the culture period [41]. For that study, adipogenic induction was performed either after 7, 14 or 21 days of culture with AsA and cell sheets were superposed at day 28 to create thicker tissues.

### Histological analyses and adipocyte surface area measurements

Samples of native and reconstructed AT (three layers-thick) were formalin-fixed and paraffin embedded. Cross sections (5 μm) were stained with Masson’s trichrome and pictures taken using a microscope Nikon Eclipse Ts100 equipped with a Nikon Coolpix 4500 camera (Nikon, Montreal, Qc, Canada). Histology micrographs were analyzed to determine the mean area (μm^2^) occupied by individual adipocytes on tissue sections using a semi-automated protocol outlining adipocyte contours (Simple PCI software, Hamamatsu Corporation, Bridgewater, NJ). The number of adipocytes per μm^2^ of tissue and their surface area were determined in order to generate mean values and frequency distribution graphs for each time-point examined. On average, more than 900 adipocytes were measured for each sample (n = 3–4) at each time-point.

### Immunolabelings and imaging of whole mount tissues

Confocal imaging on whole mount samples of reconstructed (56 days of culture/49 days of differentiation) and native AT obtained from lipectomy procedures (37 and 51 year-old donors, BMI < 25) were performed using modifications of previously described methods [42]. Briefly, ~ 8 mm^3^ free-floating formalin-fixed samples were washed in 1% w/v bovine serum albumin (BSA, Sigma) in PBS before incubation at 4°C for 48 h with either a polyclonal rabbit anti-human collagen type IV primary antibody (Ab6586 (GR696641), 2.5 μg/ml, Abcam inc, Toronto, ON, Canada) or polyclonal rabbit IgG (AB-105-C (ER1211041), 2.5 μg/ml, R&D systems, Minneapolis, MN, USA) as negative control. Samples were then incubated in a 1% PBS-BSA solution containing goat anti-rabbit IgG-coupled Alexa633 secondary antibody (A-21071 (1073050), 5 μg/ml, Molecular Probes) for 72 h at 4°C. In order to visualize the lipid content of formalin-fixed AT samples, incubation for a minimum of 2 h was performed in a 200 ng/ml Nile Red solution (N-1142 (0151–12), Life technologies, Burlington, ON, Canada). Images were acquired using a Zeiss LSM700 scanning laser confocal microscope and image software (2011, Carl Zeiss MicroImaging GmbH, Jena, Germany). They were processed using the Zen software (Zeiss) to obtain two-dimensional (2-D) representations of initial 3-D images by applying a ‘‘Maximum Intensity Projection” method [42].

### Scanning electron microscopy (SEM)

Tissue samples were fixed with 1.25% glutaraldehyde/2% paraformaldehyde in 0.1 M cacodylate buffer for 24 h before being processed with hexamethyldisilazan followed by gold-palladium coating. All micrographs were obtained at 30 kV on a JEOL 6360LV SEM microscope (Tokyo, Japan).

### TNF-α treatments

Lyophilized recombinant human TNF-α (EMD Millipore (Merck) /Cedarlane, Burlington, ON, Canada) was reconstituted in water, aliquoted and stored at -20°C. At the time of treatment, serial dilutions were prepared and added to the wells at a final concentration of 10 or 100 ng/ml TNF-α. Human reconstructed connective and adipose sheets (27 days of adipogenic differentiation) were assessed in triplicate from one (6 h, 72 h) or two distinct experiments (24 h, two different cell populations). Twenty-four hours before the treatment, the cultures were changed to DH medium supplemented with 10% FCS and antibiotics. Connective and adipose sheets were incubated with TNF-α for specified concentrations and durations before being harvested, washed in PBS, quick-frozen in liquid nitrogen and stored at -80°C for mRNA analysis. Conditioned media were also harvested at indicated time-points and stored at -80°C for analysis. For explants, AT fragments obtained after lipectomy procedures (average weight 515.9 mg, Table 1) were exposed or not to 10 ng/ml of TNF-α for 24 h in 24-well plates containing 1.0 ml of adipocyte maintenance medium devoid of dexamethasone. Explants were incubated at 37°C in a humidified atmosphere containing 8% CO_2_. Controls for protein quantification consisted of media alone incubated under the same conditions. Conditioned media were harvested and frozen at -80°C until analysis.

### Quantitative Real-Time PCR method

Total RNAs were isolated from engineered cell sheets treated with TNF-α (10 or 100 ng/ml) or their correspondent control media for 6 h (n = 3), 24 h (n = 6, 2 distinct experiments) or 72 h (n = 3). Tissues were homogenized in Qiazol buffer (Qiagen, Germantown, MD, USA) and total RNA was extracted using the RNeasy mini kit on-column DNase (Qiagen, Hilden, Germany) treatment following the manufacturer’s instructions. Quantity of total RNA was measured using a NanoDrop ND-1000 Spectrophotometer (NanoDrop Technologies, Wilmington, DE, USA) and total RNA quality was assayed on an Agilent BioAnalyzer 2100 (Agilent Technologies, Santa Clara, CA, USA). First-strand cDNA synthesis was accomplished using 2.8–4.5 μg of isolated RNA in a reaction containing 200 U of Superscript III Rnase H-RT (Invitrogen Life Technologies, Burlington, ON, Canada), 300 ng of oligo-dT_18_, 50 ng of random hexamers, 50 mM Tris-HCl pH 8.3, 75 mM KCl, 3 mM MgCl_2_, 500 μM deoxynucleotides triphosphate, 5 mM dithiothreitol, and 40 U of Protector RNase inhibitor (Roche Diagnostics, Indianapolis, IN, USA) in a final volume of 50 μl. Reaction was incubated at 25°C for 10 min, then at 50°C for 1 h and PCR purification kit (Qiagen) was used to purify cDNA.

Oligoprimer pairs were designed by GeneTool 2.0 software (Biotools Inc, Edmonton, AB, Canada) and their specificity was verified by blast in the GenBank database. The synthesis was performed by IDT (Integrated DNA Technology, Coralville, IA, USA) (Table 2). cDNA corresponding to 20 ng of total RNA was used to perform fluorescent-based Realtime PCR quantification using the LightCycler 480 (Roche Diagnostics, Mannheim, Germany). Reagent LightCycler 480 SYBRGreen I Master (Roche Diagnostics, Indianapolis, IN, USA) was used as described by the manufacturer with 2% DMSO. The conditions for PCR reactions were: 45 cycles, denaturation at 95°C for 10 sec, annealing at 57–62°C for 10 sec, elongation at 72°C for 14 sec and then 74°C for 5 sec (reading). A melting curve was performed to assess non-specific signal. Calculation of the number of copies of each mRNA was performed according to Luu-The et al., using a second derivative method and a standard curve of Cp versus logarithm of the quantity [43]. The standard curve was established using known amounts of purified PCR products (10, 10^2^, 10^3^, 10^4^, 10^5^ and 10^6^ copies) and a LightCycler 480 v1.5 program provided by the manufacturer (Roche Diagnostics). PCR amplification efficiency was verified. Normalization was performed using the geometric mean data from three reference genes shown to be genes having stable expression levels in adipose-derived stem cells and adipose tissue: glucuronidase, beta (GUSB), ubiquitin C (UBC), TATA box binding protein (TBP) [44]. Quantitative Real-Time PCR measurements were performed by the CHU de Québec Research Center (CHUL) Gene Expression Platform, Québec, Canada and were compliant with MIQE guidelines [45].

**Table 2:** Primer sequences and gene description.

Gene Symbol	Description	GenBank	size (pb)	Primer sequence 5'→3' S/AS
***CCL2***	Homo sapiens chemokine (C-C motif) ligand 2 (CCL2)	NM_002982	188	GCAGCCACCTTCATTCCCCAA/GCACAGATCTCCTTGGCCACA
***SLC2A4***	Homo sapiens solute carrier family 2 (facilitated glucose transporter), member 4 (SLC2A4)	NM_001042	212	CCAGTATGTTGCGGAGGCTAT/CGTTCTCATCTGGCCCTAAATACT
***LIPE***	Homo sapiens lipase, hormone-sensitive (LIPE)	NM_005357	191	GAAGACTCTGCAGGGATCCAATA/TTTGGATGTAAGGTGATTGCTGTGG
***FASN***	Homo sapiens fatty acid synthase (FASN)	NM_004104	257	TGCGTGGCCTTTGAAATGTGCT/ACACGCTCCTCTAGGCCCTTCA
***NFKB1***	Homo sapiens nuclear factor of kappa light polypeptide gene enhancer in B-cells 1 (NFKB1), 2 transcripts	NM_003998	180	AGCCTCTCTATGACCTGGATGACT/GCTGTTTCATGTCTCCTTGTGCTAGT
***NFKB2***	Homo sapiens nuclear factor of kappa light polypeptide gene enhancer in B-cells 2 (p49/p100) (NFKB2), 4 transcripts	NM_001077494	256	ACGAACAGCCTTGCATCTAGCC/CCCTTCAGAGTCCGAGTCGCT
***NFKBIA***	Homo sapiens nuclear factor of kappa light polypeptide gene enhancer in B-cells inhibitor, alpha (NFKBIA)	NM_020529	143	TACCTGGGCATCGTGGAGCTT/TCAGCCCCACACTTCAACAGGA
***IKBKB***	Homo sapiens inhibitor of kappa light polypeptide gene enhancer in B-cells, kinase beta (IKBKB), 8 transcripts	NM_001556	215	GCCGTGAGAAAAGTGCTTGGAG/GGCCGCAACTATAATTAAACTGTCTG
***TRAF1***	Homo sapiens TNF receptor-associated factor 1 (TRAF1), 3 transcripts	NM_005658	244	AACCCATCTGTCGCTCTTCATC/GTAGGCGTGCTTGGGTGACTG
***TNFAIP3***	Homo sapiens tumor necrosis factor, alpha-induced protein 3 (TNFAIP3), 3 transcripts	NM_001270508	241	GGCCTCTTTGATACACTTTTGCT/ACCATCACAAAAGGCCACATCT
***BIRC3***	Homo sapiens baculoviral IAP repeat containing 3 (BIRC3), 2 transcripts	NM_001165	171	TTCATCCGTCAAGTTCAAGCCAGT/CACGGCAGCATTAATCACAGGA
***BCL2***	Homo sapiens B-cell CLL/lymphoma 2 (BCL2), 2 transcripts	NM_000633	98	GGTGGGGTCATGTGTGTGGAGAG/TGCAGGTGCCGGTTCAGGTACT
***JUN***	Homo sapiens jun proto-oncogene (JUN)	NM_002228	229	CGGCCAACATGCTCAGGGAAC/ACCCTTGGCTTTAGTTCTCGGACAC
***UBC***	Homo sapiens ubiquitin C (UBC)	NM_021009	127	CTCGGCCTTAGAACCCCAGTA/AGAATCGCCGAGAAGGGACTAC
***GUSB***	Homo sapiens glucuronidase, beta (GUSB)	NM_000181	130	CGACGAGAGTGCTGGGGAATA/TTGGCTACTGAGTGGGGATACCT
***TBP***	Homo sapiens TATA box binding protein (TBP), 2 transcripts	NM_003194	189	CGGGCACCACTCCACTGTATC/GCTTGGGATTATATTCGGCGTTTC
**gDNA (Control)**	Homo sapiens 3-beta-hydroxysteroid dehydrogenase/delta-5-delta-4-isomerase (3-beta-HSD) gene (intron)	M38180	260	GAAGGGCAGAGGTGGAACTAGAA/AACAAAGACCAAAGACCAGTGAGA

### Adipokine quantification by ELISA assays

Complete culture media conditioned for 48 h by connective or adipose cell sheets were harvested each week during 7 weeks and stored at -80°C before Ang-1 analyses (Duoset®, R&D systems). Results are presented for cells originating from three different donors and are expressed as ng/ml ± standard deviation (SD). Also, connective cell sheets or adipose sheets featuring adipocytes differentiated for 28 days were treated or not with 10 or 100 ng/ml of TNF-α for 24 h. Conditioned media (n = 6 per experimental group, mean ± SD) were analyzed for MCP-1 (Duoset® ELISA, R&D systems), free NGF (Emax® ImmunoAssay Systems, Promega, Madison, WI, USA), HGF, VEGF and leptin (all DuoSet® from R&D systems). For the comparative study of the secretion profiles, media conditioned by AT explants, hrCT and hrAT (detailed in Table 1) were harvested and frozen at -80°C until analysis. Leptin, Ang-1, VEGF, PAI-1 (DuoSet®, R&D systems) and HGF levels were then quantified. Reconstructed tissues cultured for 35 days (hrCT) and 28 days of adipogenic differentiation (hrAT) were used and their supernatants harvested by collecting the complete serum-containing 48 h-conditioned media (20–22 ml). Finally, for each molecule and experiment, controls were performed by incubating the appropriate media in absence of cells, and if applicable, by subtracting the baseline levels from data.

### Determination of DNA content

In order to account for the different weights and cellularity of AT explants compared to hrAT, data normalization was performed using total DNA content of the corresponding AT explants. For hrCT and hrAT, tissues reconstructed from 4 cell populations (N = 4, n = 2–3) were used for paired data normalization. DNA content was determined using the Quant-iT™ PicoGreen® dsDNA Assay Kit (Life Technologies). Briefly, tissues were digested overnight at 56°C in 10% Proteinase K solution (Qiagen). The remaining lipid phase was discarded and the aqueous phase was treated with RNAse A (Life Technologies) for 2 h. As per manufacturer instructions, the Picogreen® reagent was added to diluted samples and _λ_DNA standards and incubated for 5 minutes before reading (Excitation: 485 nm; Emission: 520 nm) using a Varioskan Flash multimode reader (Thermo Electron Corporation) and SkanIt RE for Varioskan Flash 2.4.3 software.

### Oil Red O staining and lipid quantification

Adipose cell sheet engineering was performed as detailed above in presence or not of the wave-like movement (GyroTwister™ and Ocelot Rotator, Fisher Scientific). Lipid quantification was performed as previously described [39] on adipose cell sheets that were induced to differentiate into adipocytes at d7, d14 and d21 of culture, and further cultured for a fixed period of 14 days of differentiation. The corresponding connective sheets engineered from the same cells without adipogenic induction and cultured for the same amount of time were used as controls. Results are expressed as relative units corresponding to the mean of the ORO values (± standard error of the mean, SEM) obtained for the cultures induced with the adipogenic cocktail in reference to the OD reading of the non-induced cultures. Seven distinct experiments were performed using cells from 4 different donors. Each experiment was performed in triplicate. Oil red O staining was also performed on transverse cryosections (20 μm) of formalin-fixed tissues embedded in optimal-cutting temperature (OCT) compound. Staining of the tissues for 15 min was followed by rinsing in PBS buffer before photographs being taken with a Nikon Eclipse Ts100 microscope with a Nikon Coolpix 4500 camera.

### Statistical analyses

Data are expressed as mean ± SD for representative experiments, and as mean ± SEM when presenting results pooled from multiple experiments/donors. Statistical comparisons were made using the GraphPad Prism 6 software (GraphPad Software Inc., La Jolla, CA, USA) and are specified in the figure legends (one-way analysis of variance (ANOVA), one-sample *t*-tests or unpaired *t*-tests). The confidence interval was set at 95% (*P*≤0.05).

## Results

### Reconstruction of metabolically active human adipose tissue

**Fig 1:**
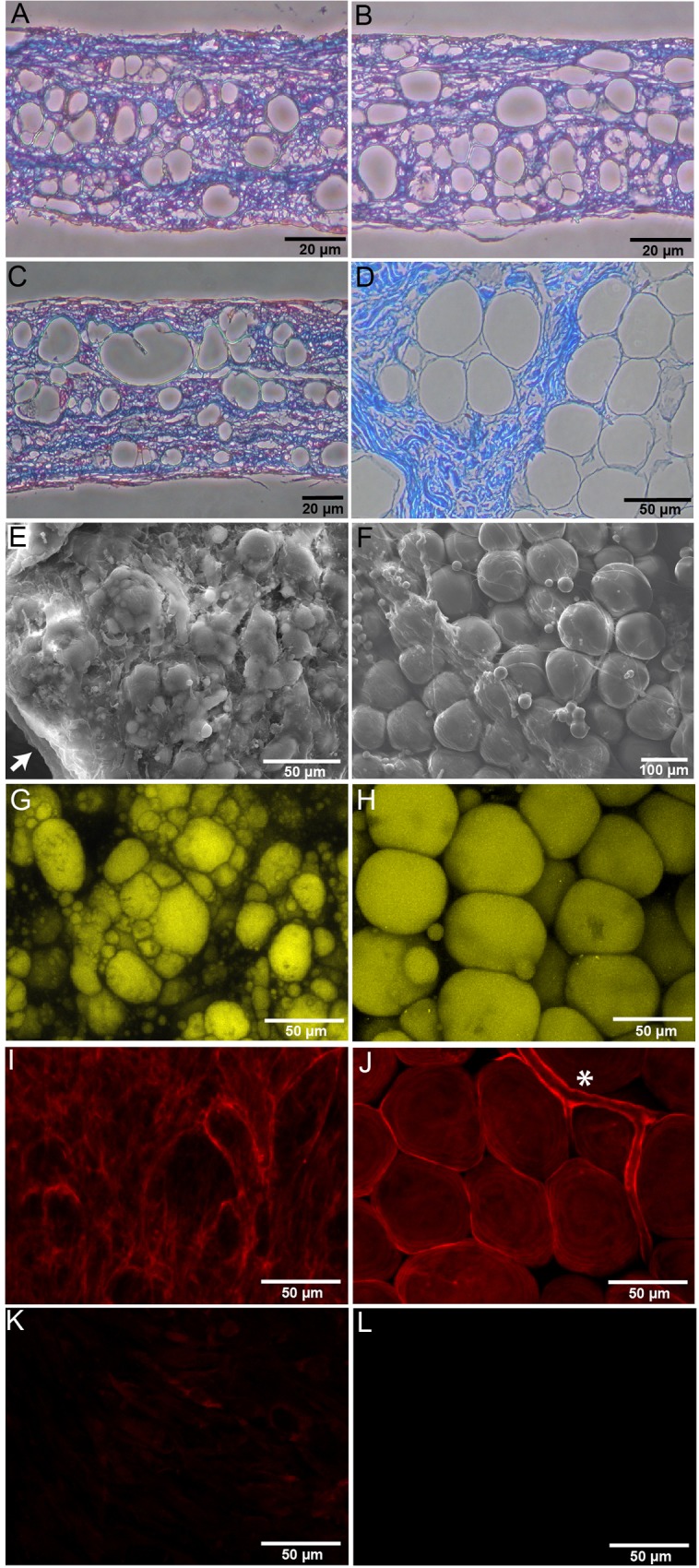
Morphological features of hrAT. The structural appearance of the hrAT engineered *in vitro* is revealed on tissue cross-sections after Masson’s trichrome staining. (A-C) Numerous adipocytes embedded in ECM can be distinguished within tissues submitted to adipogenic induction after 7 days of culture and differentiated for (A) 28, (B) 49 and (C) 81 days. (D) Histology of human native subcutaneous AT from a 54 year-old donor. (E) Scanning electron microscopy exposes rounded adipocytes (25 days of differentiation) in the matrix-rich hrAT. The arrow points to a region of the hrAT revealing the sheet-like structure. (F) Appearance by SEM of adipocytes from a 58 year-old donor. Lipids within adipocytes from (G) hrAT and (H) AT samples can be observed after Nile Red staining. Staining for collagen type IV on (I) hrAT and (J) native AT reveals its localization in basement membranes. (K) and (L) represent isotype antibody controls for labeling of hrAT and native AT, respectively. Asterisk (*): blood capillary.

Highly natural and manipulatable hrAT were produced *in vitro*. They exhibited features of human WAT at the histological (Fig 1A, 1B and 1C) and scanning electron microscopy (SEM) levels (Fig 1E). Masson’s trichrome staining was performed on sections of paraffin-embedded reconstructed tissues maintained in culture for up to 88 days. Adipogenic induction was performed after 7 days of culture with AsA-containing medium, and adipocyte differentiation was allowed to progress for 28 (Fig 1A), 49 (Fig 1B) and 81 days (Fig 1C), respectively. Histological cross-sections reveal numerous adipocytes (void spaces) embedded into the cell-derived ECM (in blue). The latter is more homogenously distributed within hrAT than for native AT (Fig 1D), for which adipocytes are arranged into fat lobules supported by dispersed ECM-rich stroma. Low magnification overview by SEM highlights the varied range in diameter of the *in vitro* differentiated adipocytes of the reconstructed tissue (Fig 1E), as well as the cell sheet structure (Fig 1E, arrow). A similar topology was observed for AT samples that features a greater proportion of larger adipocytes (Fig 1F). The lipid content of the adipocytes is revealed after staining with Nile Red dye (Fig 1G and 1H). Adipocytes are surrounded by a basement membrane containing collagen type IV, both for reconstructed (Fig 1I) and native AT (Fig 1J). Collagen type IV is also present in the basement membrane of blood vessels of native AT samples (Fig 1J, asterisk).

**Fig 2:**
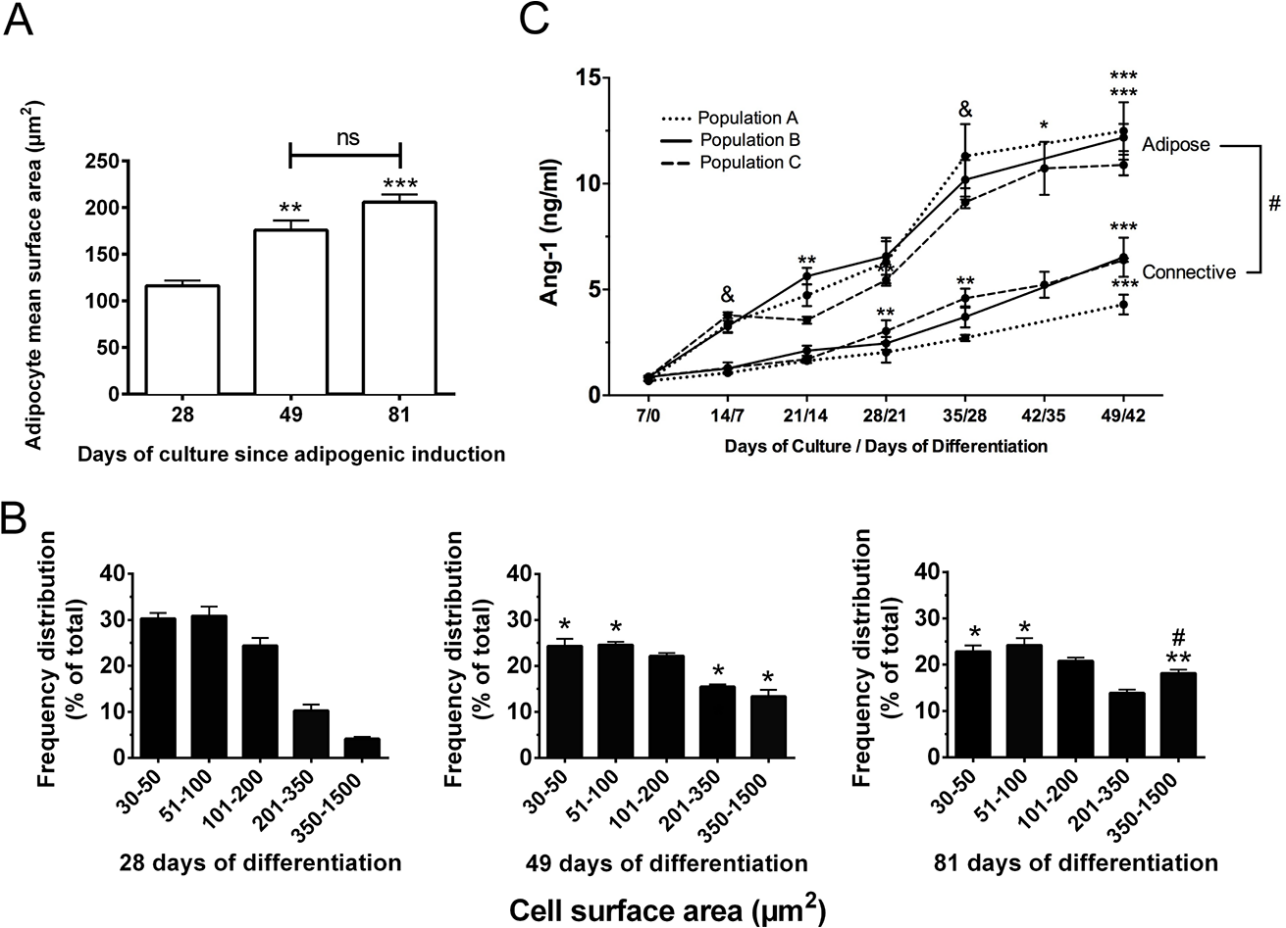
Long-term stability of the hrAT *in vitro*. (A) Mean surface area of the adipocytes over the culture period as measured from histological sections of hrAT harvested after 28, 49 or 81 days of *in vitro* differentiation. (B) Frequency distribution of adipocyte cell surface area according to the number of days the tissues were maintained in culture after adipogenic induction. Mean ± SEM. One-way ANOVA followed by Tukey’s post-hoc tests were performed and the significance is indicated in reference to day 28 (*) or day 49 (#). ***P≤0.001, **P≤0.01, * and ^#^ *P*≤0.05. (C) Kinetics of Ang-1 secretion in media conditioned by reconstructed cell sheets maintained in culture up to 49 days. Connective sheets were produced using the same cells as adipose sheets but without the adipogenic induction step. Results are expressed as ng/ml/48 h per sheet of 3.5 cm^2^. Data from cell sheets engineered from three distinct cell populations is represented (N = 3, n = 2–3 for each time-point). Mean ± SD, # indicates statistical difference between connective and adipose sheets at each time-point (*P*≤0.05, unpaired *t*-tests) while asterisks (*) indicate the comparison between consecutive weeks within the same cell population. & indicates that all three populations are significantly different between consecutive weeks. One-way ANOVA followed by Tukey’s post-hoc test. **** *P*≤0.0001, *** *P*≤0.001, **P≤0.01, * and ^&^ *P*≤0.05.

The hrAT produced were not only structurally stable over a long culture period but were continuously metabolically active and responsive to the culture environment. This is supported by the mean adipocyte size increase over time in culture (Fig 2A), as measured from adipocyte surface area on histological cross-sections, reflecting the progressive accumulation of lipid droplets. In particular, frequency distribution graphs indicate a gradual decrease in the number of adipocytes featuring smaller surface areas at both 49 days and 81 days of adipogenic differentiation, while larger adipocytes of 350–1 500 μm^2^ increased almost 4.5 times at the end of the culture period, namely 81 days after adipogenic induction (Fig 2B). In addition to this capacity to expand in size and store triglycerides, adipocytes within the reconstructed tissues actively secreted bioactive molecules. Increasing amounts of the angiogenic modulator Ang-1 (Fig 2C) were quantified in the media conditioned by the reconstructed sheets along with adipocyte development (up to 42 days of differentiation). Connective sheets produced from the same cells without adipogenic induction produced lower levels of Ang-1 at each time-point (Fig 2C), indicating a contribution of undifferentiated stromal cells to the Ang-1 levels produced by adipose sheets.

### Main secretory products detected in the conditioned media: a comparison with AT explants

**Fig 3:**
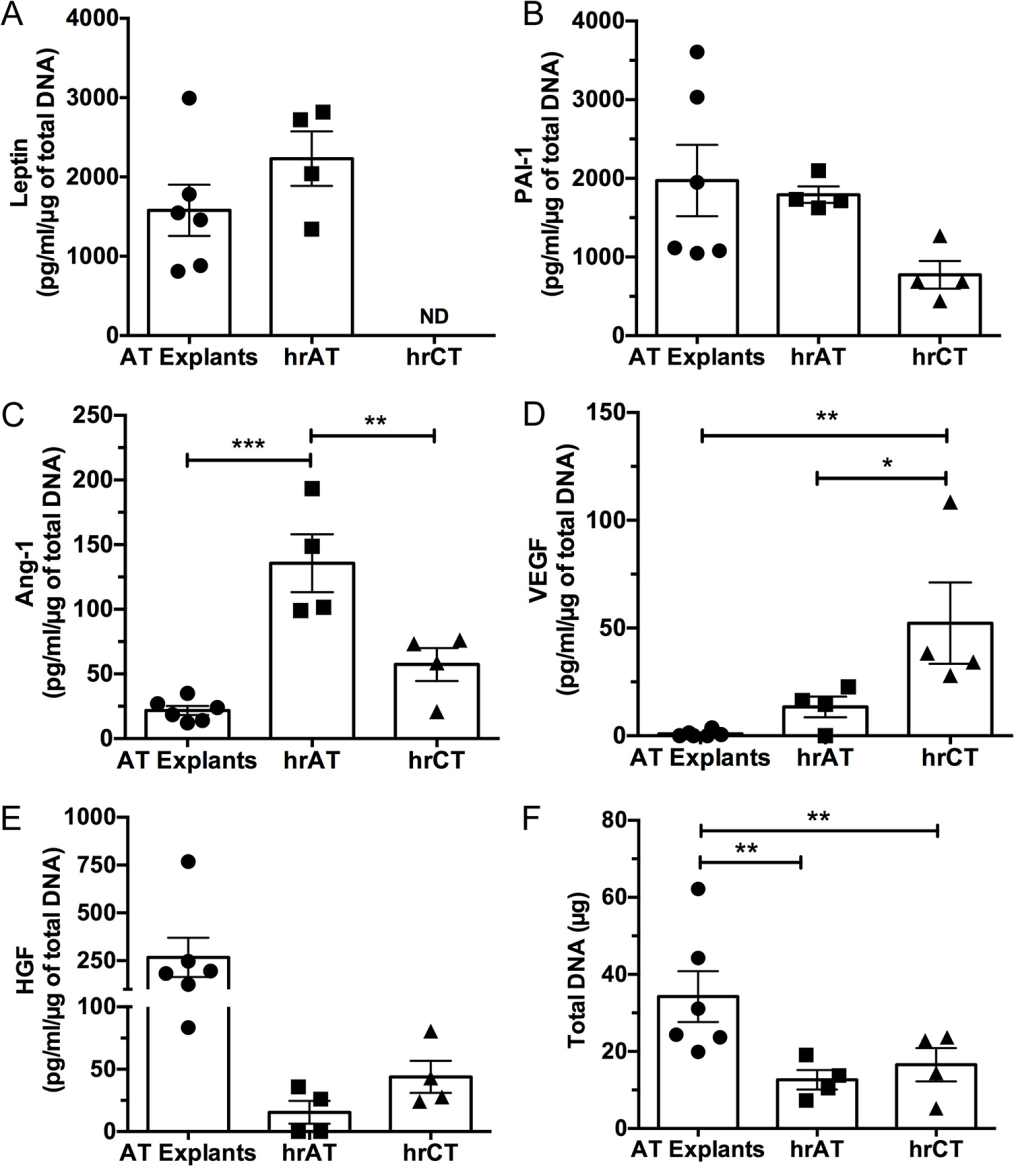
Secretion of key adipokines by AT explants and reconstructed tissues. Culture media conditioned for 48 h by AT explants (N = 6 donors, n = 3–6 per donor) as well as hrAT (28 days of differentiation) and their hrCT undifferentiated counterparts (N = 4, n = 2–3) were analyzed by ELISA assays. Secreted levels of (A) leptin, (B) PAI-1, (C) Ang-1, (D) VEGF and (E) HGF were determined. Data are expressed as pg/ml of molecule secreted in 48 h normalized by total DNA content (means ± SEM). Each datapoint represents the mean value obtained for many samples derived from a distinct donor/population (Table 1). (F) Total DNA content determination. One-way ANOVA followed by Tukey’s post-hoc test. ***P≤0.001, **P≤0.01, *P≤0.05. ND = not detected.

We then assessed the secretion of the major adipokine leptin as well as a range of molecules (VEGF, Ang-1, HGF, PAI-1) reflecting the physiological functions mediated by AT such as the modulation of angiogenic processes. We quantified these molecules in the conditioned media of reconstructed tissues featuring adipocytes differentiated for 28 days in comparison to human AT explants maintained in complete adipocyte maintenance medium for 2 days after AT harvest (Fig 3 and Table 1). Data was normalized using total DNA content (Fig 3F) since weight and cellularity varied among samples, donors and tissue types. Measured amounts of leptin (Fig 3A) and PAI-1 (Fig 3B) were within the same order of magnitude between hrAT and AT explants. Higher levels of the pro-angiogenic factors Ang-1 (Fig 3C, 6.2-fold) and VEGF (Fig 3D) were noted in hrAT. In fact, almost negligible amounts of VEGF were detected in the media conditioned by the AT explants. Finally, HGF levels (Fig 3E) were more pronounced for AT explants than for hrAT or hrCT. The secretion profiles of the undifferentiated stromal cells within hrCT were also established and compared to hrAT reconstructed from the same ASC populations. On average, these connective tissues secreted comparable amounts of HGF, slightly higher amounts of VEGF (up to 3.9-fold), but lower levels of PAI-1 (2.3-fold, *t*-test, *P* = 0.0025) and Ang-1 (2.4-fold) than hrAT.

### Modulating the functional responses within engineered tissues

Adipocytes are influenced by the specific microenvironment they are sensing. Their biological responses can be modulated by external stimuli such as the pro-inflammatory cytokines TNF-α and IL-1β. We probed the *in vitro* responsiveness of the reconstructed adipose sheets to TNF-α by monitoring gene expression in the tissues and assessing important secreted factors in conditioned media. Expression of both TNF-α receptors was detected at the mRNA level (Table 3), with higher expression level of *TNFRSF1A* in comparison to *TNFRSF1B* (14-fold). *TNFRSF1A* expression was slightly decreased by TNF-α, while the expression level of *TNFRSF1B* was significantly increased (up to 3.8-fold) in presence of TNF-α (Table 3).

**Table 3:** Gene expression in adipose sheets is modulated by TNF-α exposure.

Gene symbol	Fold variation over control ^#^					
Treatment duration	6 h		24 h		72 h	
[TNF] (ng/ml)	10	100	10	100	10	100
***TNFRSF1A***	-1.1	-1.1	-1.2	-1.2	-1.0	-1.2
	*	*	**	****		***
***TNFRSF1B***	2.3	2.6	1.7	2.0	1.8	3.8
	**	*	****	****	***	**
***CCL2***	9.8	8.7	4.4	9.6	2.3	4.4
	**	***	**	****	*	
***SLC2A4***	-1.3	-1.3	-2.2	-2.5	1.0	-1.4
	*	*	**	***		*
***FASN***	-1.6	-1.5	-1.7	-2.1	1.0	-2.8
	*	*	**	***		***
***LIPE***	-2.2	-3.2	-1.8	-2.8	-1.5	-3.2
	**	***	**	****		**
***PTGES***	3.5	3.9	4.1	5.4	1.1	3.3
	**	***	**	***		**
***PTGES2***	-1.0	1.2	1.0	1.1	-1.1	1.1
		****				
***PTGES3***	1.1	1.1	1.1	1.2	1.2	1.3
			**	***		*
***NFKB1***	7.3	7.8	2.3	3.3	1.5	3.4
	**	**	***	****	***	**
***NFKB2***	7.3	9.3	3.6	5.8	1.5	4.8
	***	**	**	****	***	***
***IKBKB***	1.7	1.7	1.2	1.3	1.0	1.2
	***	*	*	****		*
***NFKBIA***	9.5	12.7	4.9	9.7	1.8	8.6
	***	**	**	****	*	**
***BIRC3***	37.1	38.7	15.8	34.6	3.0	28.6
	**	***	***	****	***	**
***TNFAIP3***	25.3	35.3	13.4	29.7	2.4	25.6
	***	**	*	****	**	**
***PTGS2***	12.2	21.7	-1.1	3.4	-1.3	1.4
	**	**		***	**	
***TRAF1***	10.0	15.0	2.9	5.6	1.3	10.5
	**	**	**	****	***	*
***JUN ***	3.0	3.3	1.2	2.1	1.3	2.3
	**	**		****		**

(-) indicates a decrease in expression.

^#^ Statistical analyses were performed for each time-point using one-sample *t*-tests comparing fold variation ratio of each TNF-α condition to the untreated controls (value of 1).

(****P≤0.0001

***P≤0.001

**P≤0.01

*P≤0.05).

**Fig 4:**
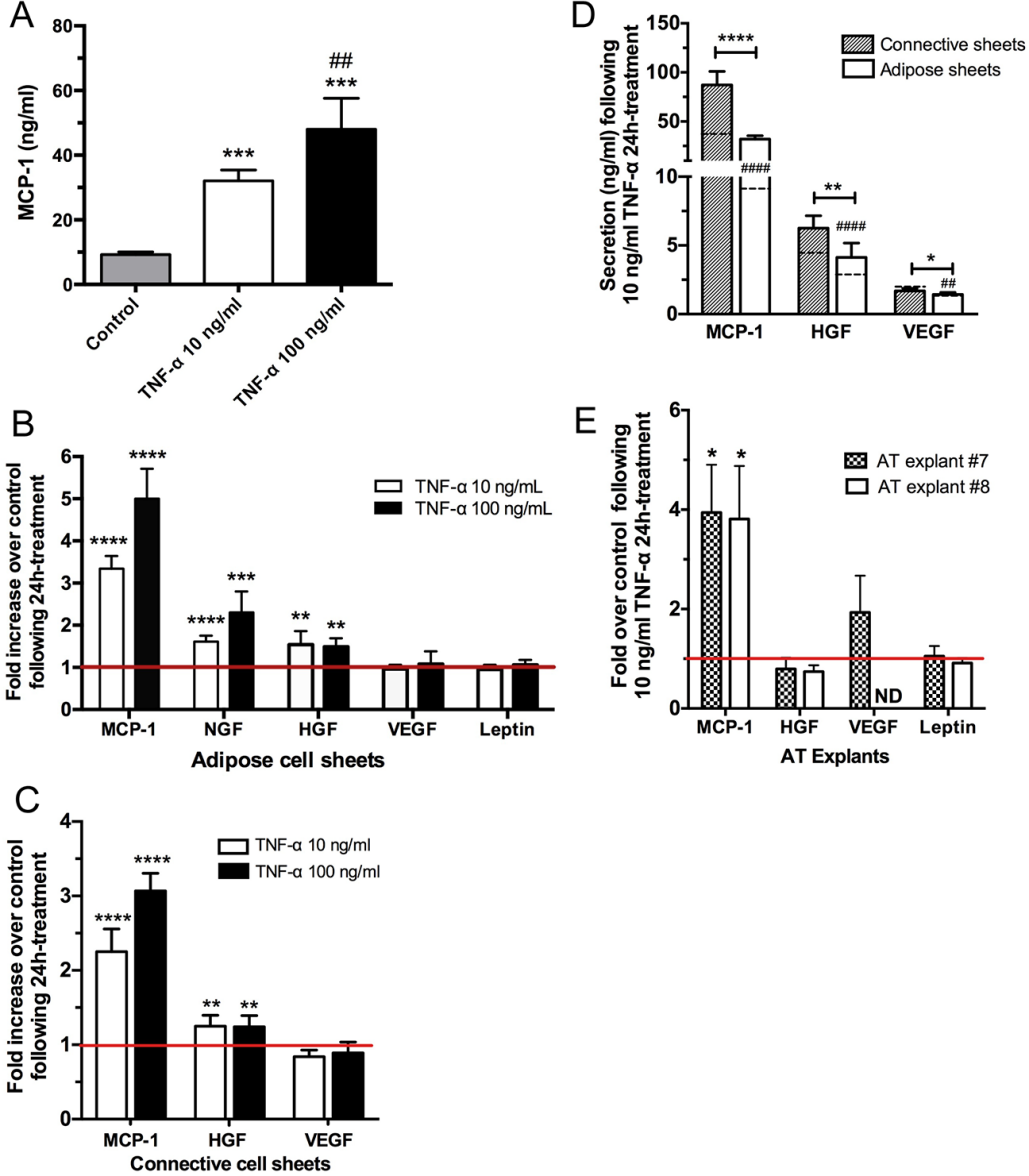
Effects of TNF-α on adipokine secretion by reconstructed tissues and AT explants. (A) Dose-dependent release of MCP-1 after exposure to TNF-α. Adipose cell sheets were incubated for 24 h in presence of 10 or 100 ng/ml of TNF-α and the conditioned media were analyzed by ELISA assays. One-way ANOVA followed by Tukey’s post-hoc test. ***P≤0.001 compared to control, ^##^ *P*≤0.01 compared to 10 ng/ml TNF-α. (B) Fold increase protein expression over control for MCP-1, free NGF, HGF, VEGF and leptin following a 24 h exposition of adipose sheets to 10 or 100 ng/ml TNF-α. (C) Fold increase protein expression over control for MCP-1, HGF and VEGF following a 24 h exposition of connective sheets to 10 or 100 ng/ml TNF-α. For each molecule, one-sample *t*-tests were performed in reference to untreated sheets (ratio of 1). For B and C, One-way ANOVA followed by Dunnett’s post-hoc test ****P≤0.0001, ***P≤0.001, **P≤0.01. (D) Comparative amounts (ng/ml) of MCP-1, HGF and VEGF secreted by the connective and adipose cell sheets following a 24 h exposure to 10 ng/ml TNF-α. Dashed lines within each column indicate the basal level of mock-treated connective and adipose sheets for the corresponding secreted protein. # indicates statistical significance for these basal levels between connective and adipose sheets while asterisks (*) indicate significance between tissue types. ****P≤0.0001, **P≤0.01, *P≤0.05. Note that leptin is not produced by connective sheets or hrCT. (E) Fold increase protein expression over control for MCP-1, HGF, VEGF and leptin following a 24 h exposition of human AT explants to 10 ng/ml TNF-α. Data normalization was performed according to the weight of the explants.

Dosage-dependent changes in the secretory capacity of adipose cell sheets were assessed for the production of monocyte chemoattractant protein 1 (MCP-1) which has an important role for immune cell recruitment at the site of inflammation *in vivo*. Increased MCP-1 secretion was detected in conditioned media 24 h after stimulation with 10 and 100 ng/ml TNF-α (Fig 4A). Additional cytokines and growth factors reportedly influenced by or mediating TNF-α actions were also evaluated (Fig 4B). As seen for MCP-1, a significant increase of free NGF (1.6- and 2.3-fold) and HGF (1.6- and 1.5-fold) proteins were observed after a 24 h stimulation at both 10 and 100 ng/ml TNF-α concentrations, respectively. However, the basal levels of VEGF and leptin detected in those conditioned media did not vary upon TNF-α exposure (Fig 4B). In parallel, connective cell sheets were also exposed to TNF-α, and the stromal cells forming these tissues responded by an increased MCP-1 secretion (Fig 4C, 2 to 3-fold) and a slightly increased HGF secretion (1.2-fold), while VEGF did not vary. When the amounts (ng/ml) of these molecules secreted by connective and adipose sheets are compared (Fig 4D), the important contribution of stromal cells of the connective sheets can be observed, both in absence and presence of exogenous TNF-α, when compared to adipose sheets (comprising adipocytes and remaining stromal cells that did not differentiate into adipocytes). Finally, the cellular responses of AT explants to TNF-α was also investigated (Fig 4E), revealing a profile similar to those of the reconstructed tissues (Fig 4B) including the modulation of MCP-1 (3.8-fold) but no change in VEGF or leptin secretion. HGF secretion was not significantly modified by 10 ng/ml TNF-α in these AT explants (Fig 4E).

### TNF-α mediated modulation of gene expression

The earlier (6 h) and prolonged (24 h, 72 h) effects of TNF-α on gene expression of human adipocyte-abundant genes as well as on members/target genes of the NF-κB activation pathway were also examined (Table 3). Genes coding for MCP-1 (*CCL2*) as well as the metabolically relevant proteins facilitated glucose transporter 4 (Glut-4, *SLC2A4*), fatty acid synthase (FAS, *FASN*) and hormone-sensitive lipase (HSL, *LIPE*) were evaluated after exposure to 10 and 100 ng/ml TNF-α. Such stimulation led to up to 10-fold increases in gene expression for *CCL2* in adipose cell sheets after 6 h and 24 h (Table 3). In contrast, *SLC2A4*, *FASN* and *LIPE* were significantly downregulated, in particular at 100 ng/ml of TNF-α at all time-points examined (Table 3).

The prostaglandin E synthases (*PTGES*, *PTGES2*, *PTGES3*) gene expression levels were also assessed after TNF-α exposure. While a moderate increase (up to 5-fold) of *PTGES* was generally observed in presence of TNF-α, none to slight modulations (up to 1.3-fold) were detected for *PTGES2* and *PTGES3*. In addition, mRNAs encoding two DNA-binding subunits of NF-κB (*NFKB1* and *NFKB2*) were induced by TNF-α, presenting significant increases at all time-points with a more pronounced effect after 6 h (8- and 9-fold respectively) and decreasing afterwards. A much lower increase (1.2- to 1.7-fold) was observed for the IKK-β (*IKBKB*) member of the IκB kinase superfamily at 6 and 24 h for both concentrations, while only 100 ng/ml TNF-α elicited changes after 72 h. Finally, TNF-α mediated changes in the expression levels of additional NF-κB-dependent genes and genes implicated in NF-κB activation were determined. A robust *BIRC3* (cIAP2) and *TNFAIP3* induction was observed and maintained over time at 100 ng/ml TNF-α. The early significant induction observed at 6 h for *PTGS2* (COX-2) was rapidly decreased after 24 and 72 h. The 10-fold range increase at 6 h observed for *NFKBIA* and *TRAF1* decreased over time at 10 ng/ml TNF-α but was more stable over time at the higher TNF-α dose. Finally, the expression level of the *JUN* component of the transcription factor activator protein-1 (AP-1) was most efficiently increased by 100 ng/ml TNF-α concentrations at later time-points.

TNF-α mediated changes were also observed for connective sheets (Table 4). The gene expression of both TNF-α receptors and the prostaglandin E synthases (*PTGES*, *PTGES2*, *PTGES3*) were modulated in a similar fashion than for adipose sheets. The expression levels of NF-κB-dependent genes and genes implicated in NF-κB activation were also upregulated (Table 4).

**Table 4:** Gene expression in connective sheets is modulated by TNF-α exposure.

Gene symbol	Fold variation over control ^#^					
Treatment duration	6 h		24 h		72 h	
[TNF] (ng/ml)	10	100	10	100	10	100
***TNFRSF1A***	-1.1	-1.1	-1.3	-1.4	-1.1	-1.6
		*	**	**		***
***TNFRSF1B***	1.8	1.7	1.8	2.1	1.6	4.5
	*	**	**	****	*	***
***CCL2***	4.9	4.8	5.1	8.6	2.3	9.9
	**	***	***	****	*	***
***PTGES***	3.2	3.3	4.4	6.9	1.3	10.4
	*	**	**	***		**
***PTGES2***	1.0	1.2	-1.1	-1.1	-1.2	-1.2
		*	*			**
***PTGES3***	1.1	1.0	-1.2	-1.1	1.0	-1.0
			**			
***NFKB1***	4.4	4.5	2.1	3.5	1.4	3.1
	***	**	****	****	*	**
***NFKB2***	4.0	5.2	2.8	4.5	1.7	4.9
	**	**	****	***	*	**
***IKBKB***	1.5	1.5	1.1	1.1	-1.0	1.2
	**	*		**		**
***NFKBIA***	5.3	6.7	4.4	8.4	1.8	7.6
	***	**	**	****	*	**
***BIRC3***	9.8	11.2	13.3	26.7	4.0	30.3
	**	**	**	***	*	**
***TNFAIP3***	12.1	15.5	5.0	12.8	2.2	19.9
	**	**	**	***	*	**
***PTGS2***	7.7	13.4	5.9	12.0	1.1	12.9
	*	*	*	***		**
***TRAF1***	8.1	9.5	2.9	4.8	1.2	3.3
	*	**	*	****		**
***JUN ***	1.4	1.5	-1.3	-1.2	-1.1	-1.1
	*	*	****	*		

(-) indicates a decrease in expression.

^#^ Statistical analyses were performed for each time-point using one-sample *t*-tests comparing fold variation ratio of each TNF-α condition to the untreated controls (value of 1).

(****P≤0.0001

***P≤0.001

**P≤0.01

*P≤0.05).

### Generating adipocytes at different stages of differentiation

**Fig 5:**
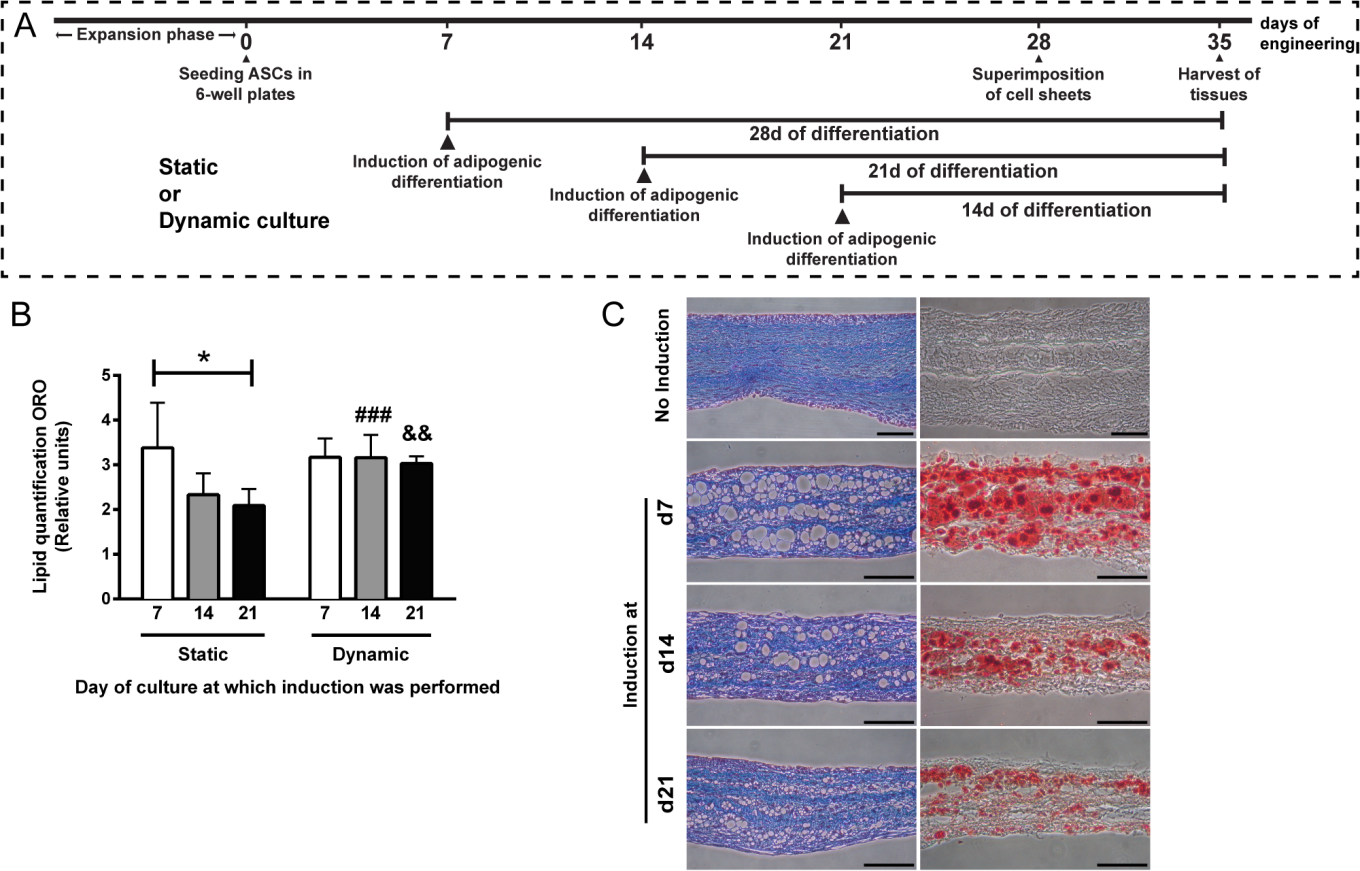
Engineering of hrAT featuring adipocytes representative of various stages of differentiation. (A) Schematic representation of the induction schemes leading to the production of the hrAT shown in (C). (B) Intracellular lipid quantification following Oil Red O staining of adipose sheets reconstructed according to static or dynamic culture conditions. While the induction of adipogenesis was performed at different times (day 7, 14 or 21) of culture in presence of AsA, Oil Red O staining was carried out after a fixed period of 14 days during which lipid accumulation proceeded. *P≤0.05, One-way ANOVA followed by Tukey’s post-hoc test; ^###^ *P* = 0.0003, ^&&^ *P* = 0.0013, paired *t*-tests between dynamic and static conditions at a given day of induction. (C) Histological cross-sections of hrCT (no adipocytes) and hrAT featuring smaller or more developed adipocytes according to the day at which induction of adipogenesis was performed under dynamic culture conditions. Masson’s trichrome staining on paraffin-embedded hrAT samples (left) show the presence of numerous adipocytes (void spaces) and important ECM content (blue), while Oil Red O staining (right) on formol-fixed cryosections reveals the accumulation of intracellular lipids by the developing adipocytes. Bars = 100 μm.

Finally, we adapted the culture conditions of our tissue engineering strategy in order to produce a wider range of hrAT featuring adipocytes at various stages of the differentiation process. The generic engineering approach consists of inducing adipogenesis after 7 days of culture with AsA, lifting the cell sheets after 28 days of culture, followed by an additional week in culture to favor cohesion between cell sheets before analysis (Fig 5A). We relied on dynamic culture conditions based on the use of a 3D rotator creating a wave-like movement on the engineered cell sheets to circumvent a particularity associated with the production of adipose tissues using the self-assembly method. While long-term ascorbate-stimulated ECM production is needed to ensure the production of manipulatable adipose sheets under static conditions, such ECM, when abundant, also reduces efficient induction of adipogenesis at later stages of culture. This is seen by the 27% (day 14) and 42% (day 21, *P*≤0.05) reduction of total intracellular lipids quantified after ORO staining on whole adipose sheets cultured for a fixed period of 14 days of differentiation (Fig 5B, Static). The use of a wave-like movement of medium throughout the culture period prevented this loss in lipid accumulation seen when induction is performed later at day 14 or 21 of culture (Fig 5B, Dynamic). No impact of the dynamic rotator culture was evidenced when induction was performed at the standard day 7 of culture. Reconstructed tissues produced under dynamic conditions were assessed both as transverse sections after Masson’s trichrome staining (Fig 5C, left) and following Oil Red O staining of formol-fixed cryosections (Fig 5C, right). When all tissues are harvested and processed at a specified time-point (after 35 days of culture), numerous small adipocytes representative of earlier stages of differentiation (differentiated for 14 days) can be seen in the hrAT induced at day 21 of culture in comparison to more developed adipocytes differentiated for a period of 21 or 28 days resulting from the adipogenic induction at day 14 and 7 of culture respectively (Fig 5C).

## Discussion

*In vitro* models adequately recapituling key aspects of adipose tissue biology are needed in order to gain novel insights into the functional roles and biological responses of human adipocytes. Primary adipocyte cultures are difficult to establish considering the buoyancy conferred by the large lipid droplets preventing cell attachment to culture surfaces. While ceiling cultures can provide a mean to study adipocytes freshly isolated from various anatomic depots, dedifferentiation of adipocytes into fibroblast-like precursor cells occurs during longer culture period [46, 47]. Direct maintenance of AT fragments *in vitro* in culture media also has limitations including high variability due to limited cell viability over time in culture [28]. It has been reported that AT viability in organotypic culture could be maintained for long periods (up to 4 weeks) by incorporating AT fragments (0.5 mm diameter) into gels made of collagen type I [48, 49]. In recent years, various tissue engineering strategies combining cells and scaffolding elements have been developed, widening the spectrum of tissue substitutes available for research and forthcoming clinical applications [37]. Engineering of human adipose tissues can be performed according to various strategies based on the use of natural or synthetic biomaterials, hydrogels or collagen gels [50]. Such tridimensional AT substitutes engineered *in vitro* are advantageous over conventional monolayer culture systems, namely because the cells are surrounded by ECM components providing important mechanical and biochemical cues. When scaffolding elements consist of naturally occurring ECM, the tissue-like context that is recreated *in vitro* then closely resemble the *in vivo* microenvironment.

Our tissue engineering model, which is based on the self-assembly approach, leads to the production of physiologically relevant hrAT devoid of synthetic or exogenous scaffolding elements. These tissues feature a variety of human ECM components including collagen type IV (Fig 1), as well as fibronectin and the structural collagens type I and V [51]. These matrix components are endogenously produced by ascorbate-stimulated cells, assembled and deposited to form cell sheets *in vitro*. The size of the reconstructed tissues can be customized by the superposition of many cell sheets of the chosen surface area. We have previously shown that hrAT express transcripts for key actors of adipogenic differentiation such as PPARγ, LPL and leptin [51]. Moreover, adipocytes within hrAT mediate β-adrenergic receptor stimulated lipolysis under standard culture conditions [39]. At the protein level, leptin is secreted in increasing amounts with cell differentiation, for at least 56 days in culture after adipogenic induction [39]. The present study establishes that sizable hrAT can be produced and manipulated with forceps, their prominent stromal compartment providing mechanical support to fragile adipocytes. They were structurally stable over a long culture period while remaining metabolically active. This is particularly relevant considering the degradation rates or remodeling events associated with other types of biomaterials available for soft tissue reconstruction. Although it cannot be excluded that some dedifferentiation events could occur at the cellular level within hrAT over 11 weeks in culture, the Ang-1 secretion profiles, combined with the adipocyte size evaluation, indicate that globally, adipocytes maintained their differentiated features, secretory activity and ability to accumulate triglycerides through *de novo* synthesis over this extended culture period.

Caution should be used when attempting to compare two different culture systems such as hrAT and AT fragments maintained as organotypic cultures. Nonetheless, parallels can be drawn and the secretion of five important adipokines was established in media conditioned by AT explants and by hrAT featuring adipocytes differentiated for 28 days *in vitro*. Data normalization using total DNA content allowed a partial adjustment for differences in weight and cell numbers among samples, donors and tissue types. Similar amounts of leptin and PAI-1 were detected between AT explants and hrAT, while the latter secreted higher quantities of Ang-1 and VEGF. The very low VEGF content that we quantified in media conditioned by AT explants is consistent with the limited VEGF release measured for AT explants from obese individuals and the predominant VEGF release from nonfat cells [52].

As previously described for tissue from obese humans, release of adipokines by AT is the result of combined secretion from matrix-resident cells as well as lipid-filled adipocytes [52]. The contribution of *in vitro* differentiated adipocytes to the secreted levels of leptin and Ang-1 is particularly significant when comparing hrAT to connective tissues devoid of adipocytes (hrCT). Our results highlight that the undifferentiated ASCs populating the connective tissues are also active producers of proangiogenic factors such as VEGF and Ang-1, for which sustained levels were detected over an extended time period (at least 49 days). Surprisingly low levels of HGF were detected in media conditioned by hrCT and hrAT in comparison to AT explants. This could suggest that other cell types present in freshly harvested AT explants contribute to HGF levels, such as endothelial cells and macrophages [16, 53].

Increased adiposity is associated with local inflammation and a dysregulation of adipokine secretion that promotes the development and maintenance of a low-grade proinflammatory state contributing to the establishment of the metabolic syndrome [3]. TNF-α being a potent inducer of adipokine changes, we investigated its impact *in vitro* on reconstructed adipose and connective sheets [20, 54]. The cellular actions of TNF-α are mediated by two receptors for which we validated the presence at the gene expression level. This is in accordance with the TNFRI and TNFRII expression reported on stromal cells and adipocytes of human subcutaneous AT [55]. Moreover, the increase in *TNFRSF1B* mRNA levels we observed upon TNF-α stimulation is reminiscent of the increase in TNFRII observed in tissues from obese patients [56]. MCP-1 is produced in high amounts by immune cells such as macrophages which are found in increased numbers in AT from obese patients. This cytokine is also produced by stromal cells and adipocytes, therefore contributing to the AT inflammatory state [57]. Our results using TNF-α-stimulated adipose sheets *in vitro* revealed increased secretion of MCP-1, NGF and HGF compared to untreated controls, while no effect on VEGF or leptin levels were observed after a 24 h exposure. TNF-α-stimulated connective sheets *in vitro* also revealed an increased secretion of MCP-1 and HGF, highlighting the important contribution of stromal cells. AT explants stimulated with 10 ng/ml TNF-α displayed a secretion profile similar to adipose sheets for MCP-1 and leptin secretion. However, the slight increase in HGF secretion seen for hrAT and hrCT was not observed in these explants, a response that could be masked by the elevated amounts of HGF already produced by the AT explants compared to the reconstructed tissues (10-fold). Collectively, similar effects of TNF-α have been described using either 3T3-L1 adipocytes, human or murine isolated adipocytes or AT explants from lean or obese individuals. In fact, numerous investigations have described the pro-inflammatory effects of TNF-α through increased MCP-1, NGF as well as HGF secretion, although increased VEGF regulation have also been observed [58–61]. Investigations of the interaction between TNF-α and leptin synthesis *in vitro* provided conflicting outcomes, as discussed by Finck and collaborators [62]. While increased leptin expression after TNF-α stimulation is often reported, other studies observed reduced leptin levels in culture [48, 60, 62–64]. These seemingly divergent reports likely arise from the use of various experimental systems and highlight the importance of characterizing each newly developed culture model. Our experiments conducted on human AT explants showed that a dose of 10 ng/ml TNF-α for 24 h did not modify leptin secretion, similarly to the results observed for hrAT. Finally, the 3D microenvironment recreated by the presence of stromal cells and endogenous ECM components surrounding human adipocytes in hrAT greatly contributed to the cellular responses observed.

While leptin secretion was not modulated by a 24 h TNF-α exposure under these culture conditions, the impact of this cytokine on genes essential for adipocyte metabolic functions including energy uptake and storage was concordant with data reported for 3T3-L1 adipocytes [65]. TNF-α downregulated the expression of genes encoding Glut-4 (*SLC2A4*) and FAS (*FASN*), two proteins that are essential for insulin-mediated uptake of glucose and fatty acid synthesis, respectively. Likewise, *LIPE* expression was also downregulated, which gene encodes HSL mediating the hydrolysis of triglycerides into fatty acids, indicating that TNF-α partly suppresses genes that are essential for metabolic functions of adipocytes, including energy uptake and storage. TNF-α is well known to modulate the expression of many response genes involved in inflammation and energy metabolism through the activation of nuclear factor κB (NF-κB). Importantly, it has been previously shown in 3T3-L1 adipocytes that NF-κB is an obligatory mediator of most of the TNF-α-induced cellular responses, namely using a non-degradable NF-κB inhibitor [65]. Although gene expression studies cannot reveal the expected translocation of the NF-κB factor to the nucleus after TNF-α stimulation, mRNA levels of IκB kinase beta (*IKBKB*) were upregulated in the treated adipose and connective sheets, and could possibly enhance the phosphorylation of IκB at the protein level and the subsequent release and activation of NF-κB. As expected, the gene expression of several components of the NF-κB signalling cascades were upregulated in TNF-α stimulated tissues such as *NFKB1*, *NFKB2* and *NFKBIA*. In addition, mRNA levels of the NFκB target genes *PTGS2*, *TNFAIP3*, *TRAF1* were significantly modulated in a time-dependent manner. Taken together, these results are suggestive of an NFκB-dependent alteration of AT-associated transcripts and secreted products in our reconstructed tissues.

The reconstructed tissues we described therefore represent unique tools to investigate in a controlled manner the effects of pharmacologically active products on human differentiated adipocytes as well as compounds modulating adipogenesis from precursor cells. The versatility of our model was further emphasized by the ability to generate tissues featuring human adipocytes at different stages of differentiation. This was achieved using dynamic culture conditions generating a wave-live movement of the media in the culture dishes. Such movement does not impact cell proliferation [41], but could likely increase mass transport and the availability of the adipogenic signal to cells.

The tissue engineering model we described is particularly useful to investigate the effects of bioactive molecules on adipocytes or stromal cells apart from the influence of other cell types present in native AT. Conversely, it is also possible to sequentially add other cell types to generate more complex tissues. For example, we and others have incorporated endothelial cells into *in vitro* reconstructed adipose tissues, allowing the concomitant evaluation of angiogenic and adipogenic processes [42, 66–68]. Using such a model based on silk biomaterial and spinner flasks cultures, Bellas *et al*. reported the engineering of human adipose tissues maintaining leptin secretion for 24 weeks in culture [68]. In the future, the incorporation of immune cells to engineered models would be highly informative for immuno-adipobiology studies mimicking inflammation.

In conclusion, we successfully engineered human AT models presenting morphological and functional characteristics closely similar to human AT. In addition to revealing the relatively long stability in culture of these engineered tissues, this study establishes the basal and TNF-α-stimulated secretory capacity of the adipocytes and stromal cells, therefore allowing long-term assessment of metabolic responses *in vitro*. The availability of tissue engineered model systems that are physiologically relevant and recapitulate the complex 3D nature of adipose tissue will likely broaden their use in toxicology screening and drug development studies.
